# The measurement of water scarcity: Defining a meaningful indicator

**DOI:** 10.1007/s13280-017-0912-z

**Published:** 2017-03-15

**Authors:** Simon Damkjaer, Richard Taylor

**Affiliations:** 1 University College London Institute for Sustainable Resources, Central House, 14, Upper Woburn Place, London, WC1H 0NN UK; 2 Department of Geography, University College London, Pearson Building, Gower Street, London, WC1E 6BT UK

**Keywords:** Scarcity indicator, Scarcity metric, Storage, Water scarcity, Water scarcity indicator, Water stress

## Abstract

Metrics of water scarcity and stress have evolved over the last three decades from simple threshold indicators to holistic measures characterising human environments and freshwater sustainability. Metrics commonly estimate renewable freshwater resources using mean annual river runoff, which masks hydrological variability, and quantify subjectively socio-economic conditions characterising adaptive capacity. There is a marked absence of research evaluating whether these metrics of water scarcity are meaningful. We argue that measurement of water scarcity (1) be redefined physically in terms of the freshwater storage required to address imbalances in intra- and inter-annual fluxes of freshwater supply and demand; (2) abandons subjective quantifications of human environments and (3) be used to inform participatory decision-making processes that explore a wide range of options for addressing freshwater storage requirements beyond dams that include use of renewable groundwater, soil water and trading in virtual water. Further, we outline a conceptual framework redefining water scarcity in terms of freshwater storage.

**Electronic supplementary material:**

The online version of this article (doi:10.1007/s13280-017-0912-z) contains supplementary material, which is available to authorized users.

## Introduction

Ensuring the availability of adequate quantities of freshwater to sustain the health and well-being of people and the ecosystems in which they live, remains one of the world’s most pressing challenges (Jiménez-Cisneros et al. 2014; Rockström and Falkenmark 2015). This challenge is enshrined in the United Nations Sustainable Development Goal (SDG) 6.4 *[…to] substantially reduce the number of people suffering from water scarcity* by 2030. Water scarcity can broadly be described as a shortage in the availability of renewable freshwater relative to demand (Taylor 2009) yet a more precise description is required to define a robust quantitative metric. Such a metric would measure and evaluate progress towards reducing water scarcity and identify where and when water scarcity may occur in the future.

Here, we critically review the most widely employed measures of ‘water scarcity’ among the more than 150 indicators that have been identified (WWAP 2003; Vörösmarty et al. 2005, p. 235). We examine the evolution of these metrics as well as the data and assumptions that inform them. The central purpose of our review is to stimulate debate about how best to measure ‘water scarcity’. We expose substantial limitations in current metrics and critically examine what characteristics might define a more robust metric. Our analysis places particular priority on the characterisation of water scarcity in low-income countries of the tropics where the consequences of water scarcity are projected to be most severe (Jiménez-Cisneros et al. 2014) and where most of the global population now live (Gerland et al. 2014).

## Review of water scarcity metrics

### Water stress index (WSI)

Falkenmark and Lindh (1974) proposed one of the first quantitative links between freshwater resources and population at the Third World Population Conference in Bucharest in 1974. Formal quantification of water scarcity began, however, in the early 1980s with the development of the water stress index (WSI) explicitly linking food security to freshwater availability (Falkenmark 1986, 1989). Conceived in the context of famines taking place across the Sudano-Sahel of Africa, the WSI was originally intended to provide an early warning system to inform strategies for food self-sufficiency in light of anticipated future droughts and a growing population. The WSI has since become the most widely applied measure of water scarcity. Despite identified limitations in this metric identified in previous reviews (e.g. Savenije 1999; Chenoweth 2008; Taylor 2009; Brown and Matlock 2011; Jarvis 2013; Wada 2013; Brauman et al. 2016), the WSI continues to be applied at regional to global scales (Vörösmarty et al. 2000; Alcamo et al. 2003; Arnell 2004; Wada et al. 2011; Wada 2013; Schewe et al. 2013).

**Fig. 1:**
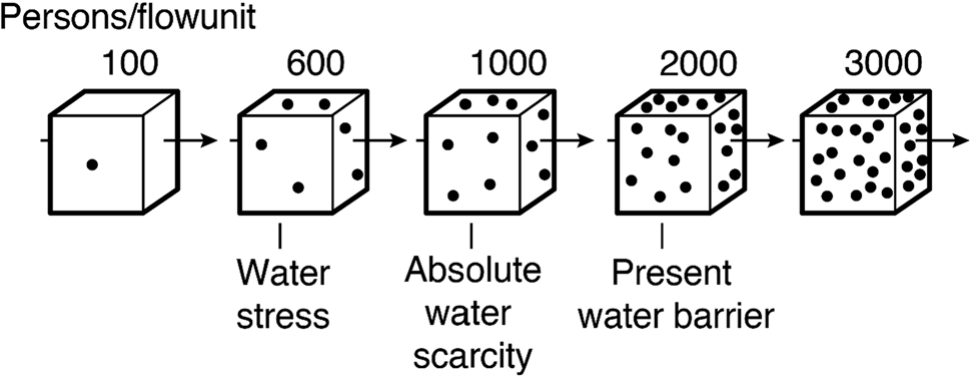
Visualisation of different levels of water competition; each cube indicates the flow of 1 million m^3^/year available in terrestrial water systems, each dot 100 individuals depending on that water (adapted from Falkenmark 1989, p. 115)

The WSI originally defined water scarcity in terms of the number of people that compete to be sustained by a single flow unit of water—defined as 10^6^ m^3^ year^−1^ (Fig. 1; Falkenmark 1986, 1989; Falkenmark et al. 1989). This “hydraulic density of population” or *une densité hydraulique de population* was considered to be a powerful instrument for demonstrating differences in water availability between countries (Forkasiewicz and Margat 1980; Falkenmark 1986). This approach was used to examine water resources availability across the globe applying readily available records of river discharge (“river runoff”) compiled by L’vovich (1979) and Forkaziewicz and Margat (1980). The basis for the threshold of water scarcity was, however, context-specific. Explicitly referring to Israel, Falkenmark (1986) argued that an industrialised country in a semi-arid zone has a gross water demand^1^ of approximately 500 m^3^ capita^−1^ year^−1^, equivalent to 2000 people/flow unit. This value was set as the threshold at the time *for operating a modern semi*-*arid society using extremely sophisticated water management* and *[…] half of this value* [1000 people/flow unit] *could be considered as relatively water*-*stressed* (Falkenmark 1986, p. 199). Falkenmark (1989, p. 115) later argued that *typical water*-*consumption levels in a number of industrialised countries are in the interval of 100*–*500 persons per flow unit*. The threshold for “water stress” in what we refer to here as the Inverted WSI (Table 1) was set at 500 people/flow unit but subsequently raised to 600 people/flow unit (~1 667 m^3^ capita^−1^ year^−1^) *in order to not exaggerate the situation* (Falkenmark 1989, p. 116); the threshold for “water scarcity” became 1000 persons/flow unit or 1000 m^3^ capita^−1^ year^−1^ (Fig. 1).

**Table 1:** Summary of water stress index thresholds

Category	Inverted WSI (people/flow units)^a^	Contemporary WSI threshold (m^3^ capita^−1^ year^−1^)
No stress	<600 people/flow unit	>1700
Water scarcity	600–1000 people/flow unit	1700–1000
Water stress	1000–2000 people/flow unit	1000–500
Absolute water stress	>2000 people/flow unit	<500

^a^A flow unit in the column for Inverted WSI is equal to 10^6^ m^3^. To get contemporary WSI, one flow unit must be divided by the number of people competing for this water

Since the conception of the WSI, different arguments have been proposed as the basis for the setting of thresholds of ‘water stress’ and ‘water scarcity’. Falkenmark (1986) originally proposed a gross per capita freshwater demand of 500 m^3^ year^−1^ that comprised a domestic and industrial demand of 50 m^3^ capita^−1^ year^−1^ [~130 L capita^−1^ day^−1^ (LCPD)] with an additional 80–90% of the per capita water demand allocated for irrigation. Domestic (household) freshwater demand was subsequently adjusted to assume 100 LCPD amounting to an annual domestic water requirement of 36.5 m^3^ capita^−1^ year^−1^ or ~40 m^3^ capita^−1^ year^−1^ (Savenije 1999). Engelman and Leroy (1993) and Gardner-Outlaw and Engelman (1997) follow a similar line of reasoning but provide a different rationale for the same thresholds outlined in Table 1. They cite Falkenmark and Widstrand (1992) to claim that agricultural, industrial and energy demands constitute 5–20 times the domestic requirement of 100 LCPD. Falkenmark and Widstrand (1992, p. 14) do not, however, specify an amount required to meet agricultural, industrial and energy demands but instead argue that in order to *[…] assure adequate health, people need a minimum of about 100* *L of water per day for drinking, cooking and washing. Of course many times this amount is necessary to carry out the activities necessary to sustain an economic base in the community*. Although what constitutes “*many times*” is not specified (Savenije 1999), Engelman and Leroy (1993) and Gardner-Outlaw and Engelman (1997) reason that by adding agricultural, industrial and energy demands (i.e. 20 times a domestic demand of 40 m^3^ capita^−1^ year^−1^) to domestic demand, a holistic water demand of 840 m^−3^ capita^−1^ year^−1^ can be computed. The authors then conclude that freshwater resources that amount to a doubling of this figure (~1700 m^3^ capita^−1^ year^−1^) provide a boundary for differentiating between *relative water sufficiency* (>1700 m^3^ capita^−1^ year^−1^) and *water stress* (<1700 m^3^ capita^−1^ year^−1^), whereas the threshold for *water scarcity* is 1000 m^3^ capita^−1^ year^−1^. These thresholds are identical to those derived differently from the inverted WSI (Table 1).

Notwithstanding the separate rationales for the thresholds of water stress and water scarcity in the WSI, the values of 1700 and 1000 m^3^ capita^−1^ year^−1^ have been uncritically adopted and assimilated in the mainstream literature without an empirical basis. For example, Chapter 4 of the Intergovernmental Panel on Climate Change (IPCC) Third Assessment Report on Hydrology and Water Resources (IPCC 2001, p. 213) states that *[…] water stress may be a problem if a country or region has less than 1700* *m* ^*3*^ *year* ^*−1*^ *of water per capita* (Falkenmark and Lindh 1976) though no such direct claim is made by Falkenmark and Lindh (1976). Similarly, Vörösmarty et al. (2005) contend that *A value of 1700* *m* ^*3*^ */capita/year* ***(20)*** *is widely accepted as a threshold below which varying degrees of water stress are likely to occur*; reference ‘20’ is the widely cited paper of Falkenmark (1989) which makes no claim to this threshold.

Early applications of the WSI (Falkenmark 1986, 1989) quantified available freshwater resources in terms of river discharge or “river runoff” equating renewable freshwater resources to mean annual river runoff (MARR). Use of MARR in the WSI has since been greatly promoted by the development of national-scale estimates of MARR based on observational records (e.g. Shiklomanov 2000) and proliferation of large-scale hydrological models estimating MARR (e.g. lcamo et al. 2003; Arnell 2004; Vörösmarty et al. 2005; Oki and Kanae 2006; Schewe et al. 2013; Wada et al. 2014), which are reconciled to national-scale and gridded population data and projections. Rijsberman (2006) and Chenoweth (2008) have investigated the links between water scarcity thresholds and indicators of national development but there remains, nevertheless, a conspicuous dearth of research assessing whether computations of water stress and scarcity based on the WSI (Table 1) are meaningful.

Use of MARR to define renewable freshwater resources implicitly assumes changes in soil moisture storage (ΔSMS) and groundwater storage (ΔGWS) are negligible, and MARR (mean annual *Q*_river_) represents the net contribution of precipitation (P) to the terrestrial water balance accounting for outflows derived from evapotranspiration (ET) (Eqs. 1 and 2). The representation of renewable freshwater resources with the singular value of MARR masks intra- and inter-annual variabilities in freshwater resources (Taylor 2009) yet such variabilities are particularly extreme in Sub-Saharan Africa (McMahon et al. 2007). Critically, MARR does not also indicate the proportion of river discharge that occurs episodically as stormflow and that which occurs throughout the year as baseflow; the latter often results from groundwater discharge. Further, MARR also does not account for soil water (“green water”) which can play a critical role in determining agricultural water demand (Rockström and Falkenmark 2015), the sector that globally accounts for the majority of freshwater withdrawals and influences the amount of available blue water resources (Jaramillo and Destouni 2015b).

1 $$ P = ET + Q_{\text{river}} + \Delta {\text{GWS}} + \Delta {\text{SMS}} $$

2 $$ \overline{{Q_{\text{river}} }} = \overline{P} - \overline{ET}  (\Delta {\text{SMS}} + \Delta {\text{GWS}} = 0) $$

### Withdrawal-to-availability ratio (WTA)

The presumption of a fixed, universal water demand, embedded in the WSI, was questioned by a second wave of water resources assessments incorporating estimates of freshwater demand both spatially and across sectors including domestic (D), industrial (I) and agriculture (A) sectors (Raskin et al. 1996). The freshwater *Withdrawal-To-Availability* (WTA) ratio defined water scarcity in terms of the ratio or percentage of total annual withdrawals across these sectors to annual (renewable) resources estimated by MARR (Eq. 3). Conducted at national scales, a country is considered ‘water stressed’ if annual withdrawals are between 20% (0.2) and 40% (0.4) of annual freshwater supply and ‘severely stressed’ if this figure exceeds 40% (0.4) (Raskin et al. 1996; Alcamo et al. 2003; Rijsberman 2006). The WTA ratio has been applied directly in numerous contexts (Table S1) and a sensitivity analysis of the 0.4 threshold ratio was carried out using the global hydrological model, WaterGAP 2.0, and declared *[…] fairly robust* (Alcamo et al. 2003) though the basis for this judgment is unclear.

3 $$ {\text{WTA}} = \frac{{\mathop \sum \nolimits {\text{DIA}}}}{\text{MARR}} $$

The use of MARR to characterise freshwater resources means that the WTA approach, like the WSI, masks seasonality and inter-annual variability in freshwater resources. The WTA approach can employ spatially and temporally variable freshwater demand functions but their estimation has their own conceptual challenges as noted by Rijsberman (2006, p. 3): *the limitations of the criticality ratio* (i.e. WTA >0.4) *and similar indicators are that: a) the data on water resources availability do not take into account how much of it could be made available for human use; b) the water withdrawal data do not take into account how much of it is consumptively used (or evapotranspired) and how much could be available for recycling, through return flows; and c) the indicators do not take into account a society’s adaptive capacity to cope with stress.* Additionally, quantified freshwater demand, transparent in the WSI, is often opaque in applications of the WTA ratio. Nevertheless, Wada (2013) contends that the WTA threshold ratio of 0.4 corresponds to the WSI threshold of 1700 m^3^ capita^−1^ year^−1^ and a category of extreme water stress is also asserted to occur at a ratio above 0.8 and equated to the WSI threshold of 500 m^3^ capita^−1^ year^−1^ though the basis for this proposed alignment of metrics is unclear.

### Emergence of holistic metrics

That measurement of water scarcity and stress may not solely be characterised by water resources but account for both: (1) the capacity of societies to adapt to different levels of freshwater availability and (2) environmental sustainability associated with freshwater use is explicitly recognised in the emergence of holistic metrics. These water scarcity metrics seek to characterise ‘adaptive capacity’ and to introduce the concept of environmental water demand in order to sustain ecosystem function. Six holistic approaches to the measurement of water scarcity are considered below.

#### Social water stress index

‘Adaptive capacity’ is explicitly considered in the social water stress index (SWSI) (Ohlsson 2000). The SWSI posits that distributional equity, political participation, and access to education are good indicators of the ability of a country to adapt to water shortages. To account for these social factors, the SWSI applies the Human Development Index (HDI) which incorporates the variables of life expectancy, educational attainment (i.e. adult literacy and combined primary, secondary and tertiary enrolment) and GDP per capita as a proxy for adaptive capacity to water shortages. The SWSI allows for the comparison of country scores between the original WSI^2^ and SWSI after adaptive capacity has been taken into account. The SWSI divides the number of people in a country that share one million cubic metres of annual renewable water (i.e. the inverted Falkenmark WSI) by the HDI (Eq. 4). The resulting value is then divided by a *scalar* which Ohlsson (2000) sets at 2. Finally, the SWSI score is compared to the HWSI score (see footnote 2), according to the rank interval classification in Table S2. Ohlsson (2000) shows how countries such as South Korea, Poland, Iran, the UK, Belgium and Peru, which are traditionally classified as water stressed according to the HWSI, would be classified as ‘relatively sufficient’ under the SWSI because of their higher societal adaptive capacity (defined by HDI). In contrast, countries that are considered to have a lower adaptive capacity such as Niger, Burkina Faso, Eritrea and Nigeria move from ‘relative sufficiency’ to ‘water stress’.

4 $$ {\text{SWSI}}_{\text{country}} = \frac{{{\text{Inverted Falkenmark WSI}}_{\text{country}} }}{{{\text{HDI}}_{\text{Country}} }}  \times \frac{ 1}{\text{scalar}} $$

Ohlsson (2000) considers the HDI to be *[…] a very appropriate and widely accepted indicator […]*. Kovacevic (2010) argues, however, that the definition of human development in the HDI is oversimplified due to its narrow selection of variables; many of these are often of low-quality data for low-income countries (Srinivasan 1994). Although metrics necessarily rely upon simplified characterisations of reality, the risk and consequences of misrepresentation, particularly in low-income countries where conditions of water scarcity may have the greatest impact, remain. Ogwang (1994) contends that the HDI does not reveal anything beyond traditional economic indicators due to the high correlation between individual components of the HDI and pure economic indicators such as gross national product (GNP) and gross domestic product (GDP).

#### Physical and economic water scarcity

**Fig. 2:**
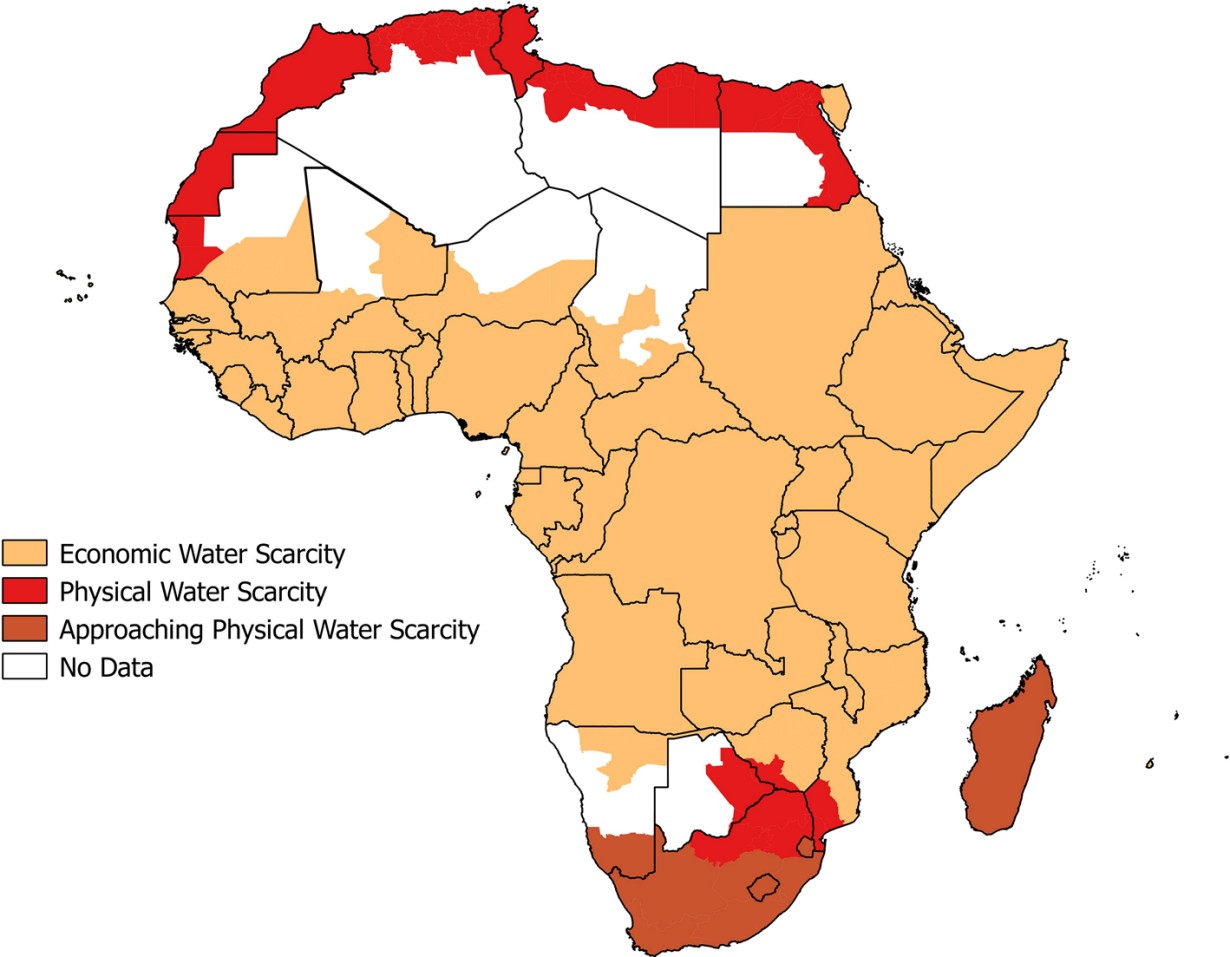
Map of physical and economic water scarcity at basin level in 2007 across the African continent, adapted/reproduced from global map Available at http://www.grida.no/graphicslib/detail/areas-of-physical-and-economic-water-scarcity_1570

The importance of adaptive capacity to the characterisation of water scarcity was highlighted by Seckler et al. (1998a) and later Molden et al. (2007) who propose future infrastructure development potential and irrigation efficiency potential (i.e. improved water management measures, return flows and consumptive uses) to be proxies of adaptive capacity. The authors then applied this measure of adaptive capacity to distinguish between ‘physically’ and ‘economically’ water-scarce countries. Physical water scarcity is said to occur in a country when more than 75% of river flows in a country are withdrawn for DIA purposes (Brown and Matlock 2011) and the country is unable to meet future demands after accounting for its adaptive capacity. Economic water scarcity is considered to occur in countries where renewable water resources are adequate (i.e. water withdrawals are less than 25% of river flows) but where there is a lack of significant investments in water infrastructure in order to make these resources available (Rijsberman 2006). The International Water Management Institute (IWMI) then mapped areas in Africa according to these criteria, which face either physical or economic water scarcity and areas expected to approach physical water scarcity (Fig. 2).

The distinction of “economic” and “physical” water scarcity appeals to reason yet both measures rely on expert judgment. Indeed, assessments of adaptive capacity through infrastructural development capacity are complicated and opaque. Seckler et al. (1998b, p. 7), for example, compiled data pertaining to infrastructural development using *secret intelligence information* via MEDEA (Measurements of Earth Data for Environmental Analysis), a group of distinguished experts who has unique access to sensitive remote-sensing information.

#### Water poverty index

The Water Poverty Index (WPI), originally proposed by Sullivan (2002), arose from a perceived need to advance the use of indicators that examine poverty in various dimensions (i.e. development, gender, food, politics, health and vulnerability) and specifically highlight the vital but overlooked links between poverty reduction and water availability. Sullivan (2002) contends that the WPI functions as a transparent and simple tool which takes a holistic approach to the representation of conditions that affect water stress at community and household levels. The WPI seeks to empower poor people to participate in water resources planning and assist decision-makers in determining priority interventions in the water sector.

The WPI employs a multi-dimensional approach that goes beyond the use of the HDI in the SWSI as a characterisation of social vulnerability and to include a measure to represent the maintenance of ecosystems (i.e. environmental sustainability). It is formed by five components *i*): (1) available water resources; (2) access to water; (3) capacity for water management; (4) water uses for domestic, food and production purposes and (5) environmental concerns. These indicators are weighted and integrated into a single measure as given in Eq. 5 where *X*_*i*_ refers to [indicator] *i* of the WPI structure for that location and *w*_*i*_ is the weight applied to that [indicator] *i*. Each *i* is made up of a number of variables that are first combined using the same technique (Fenwick 2010, p. 51). The WPI has been applied at both global (Lawrence et al. 2002) and community scales (Sullivan et al. 2006; Fenwick 2010).

5 $$ {\text{WPI}} = \frac{{\mathop \sum \nolimits_{i = 1}^{N} w_{i} X_{i} }}{{\mathop \sum \nolimits_{i = 1}^{N} w_{i} }} $$

The first component (or indicator) of the WPI, available water resources, is rooted in an estimate of per capita freshwater availability defined by the WSI (Molle and Mollinga 2003). As a result, the WPI is subject to the same limitations identified for the WSI above, including its disregard of temporal variability in water resources which plays a critical role in enabling access to a reliable amount of water (Fenwick 2010). More specifically, the WPI raises difficult questions concerning the quantification of social dimensions of freshwater availability and access. A particular challenge is the application of weights (*w*_*i*_ *)* to the various indicators (*i*) that are determined through a participatory process (Feitelson and Chenoweth 2002; Molle and Mollinga 2003; Garriga and Foguet 2010). The exercise generates locally specific results (Garriga and Foguet 2010) that restrict comparative analyses. A standard set of indicators was originally suggested to comprise the WPI (Sullivan 2002) in order to enable comparisons across space and time. However, this normalisation technique is thought to inhibit longitudinal studies (Fenwick 2010). It is also difficult to translate theoretical constructs between rural and urban settings where individual variables may not apply to both sites. The exercise of trying to quantitatively assess and compare highly subjective and relative variables such as *needs* (Fenwick 2010) becomes difficult and possibly unrealistic, given the varying perceptions and understandings of the definitions and meanings of the indicator variables. Indeed, there may be more merit to explore and discuss the individual indicators of the WPI rather than the overall water poverty score (Sullivan 2002). The WPI may thus be better suited to instigating debates around the concept of water poverty as opposed to actually measuring it, as suggested by its creators: *[…] the purpose of an index is political rather than statistical* (Lawrence et al. 2002).

#### The environment as a water user

The adoption of the Dublin Principles in 1991 whereby *effective management of water resources demands a holistic approach, which links social and economic development into the protection of natural ecosystems* explicitly recognised the water needs of the environment. This recognition promoted the inclusion of Environmental Water Requirements (EWRs) into metrics of water scarcity such as the “Water Stress Index” (WSI_EWR_) proposed by Smakhtin et al. (2004) and defined by Eq. 6. Using the WaterGAP2 model, Smakhtin et al. (2004) applied the WSI_EWR_ to a global water resources assessment and found that consideration of EWRs resulted in a greater number of basins having a higher magnitude of water stress. Further, they asserted that approximately 20–50% of MARR in different basins are required to be allocated to freshwater ecosystem in order to maintain them in a fair condition (Smakthin et al. 2004).

6 $$ {\text{WSI}}_{\text{EWR}} = \frac{\text{Withdrawals}}{\text{MARR-EWR}} $$

The assessment of an adequate amount of flow allocated for EWRs is influenced by many factors such as the size of the river, its perceived natural state and fluctuations in seasonal environmental capacities (Acreman and Dunbar 2004). Smakthin et al. (2004) showed that EWRs are the highest for rivers in the equatorial belt (e.g. parts of the Amazon and the Congo) where there is a stable rainfall input throughout the year. In areas, which are characterised by substantial monsoon-driven variability (e.g. India), EWRs are lower and generally in the range of 20–30% of MARR because aquatic biota are adapted to extended periods of limited or no flow. In contrast, stable river-flow regimes are much more sensitive to perturbations in river discharge.

Assessing EWRs ranges from objective-based methods to more holistic exercises that can involve cross-disciplinary teams providing expert judgment. The relationships among various functions of a river system are often difficult to establish with confidence and consequently require subjective judgements due to a lack of reliable hydrological, biological and ecological data in low-income countries (Acreman and Dunbar 2004). Ultimately, EWR assessments involve difficult trade-offs between environmental and human uses, and it remains unclear how best to decide, and who decides, among different uses of water.

#### Water resources sustainability

Another group of water scarcity metrics is based around the principle that water sustainability constitutes *[…] systems designed and managed to fully contribute to the objectives of society, now and in the future, while maintaining their ecological, environmental and hydrological integrity* (Loucks and Gladwell 1999, p. 30). This group of holistic metrics are ambitious, seeking to incorporate considerations of infrastructure, environmental quality, economics and finance, institutions and society, human health, welfare, planning and technology (Loucks and Gladwell 1999) as well as addressing issues such as basic water needs; minimum standard of available water resources; access to data on water resources and democratic water-related decision-making with inter and intra-generational equity in mind (Mays 2006).

The Watershed Sustainability Index (Chaves and Alipaz 2007) integrates social, economic and environmental factors under the HELP Platform of UNESCO-IHP comprising hydrology (H), environment (E), life (L) and policy (P) in Table S3; each heading has the parameters “pressure, state and response” scored subjectively at (0, 0.25, 0.5, 0.75 and 1). The score for H is the value of the WSI, whereas E relies on application of the Environment Pressure Index, a modified version of the Anthropic Pressure Index (Sawyer 1997) which is estimated from the variation in the average basin agricultural area over the variation of urban basin population. L is based on income and HDI scores, and P is determined by the HDI-Education Parameter and judgments regarding the state of IWRM in the basin.

The Canadian Water Sustainability Index (CWSI) is a composite index that evaluates the well-being of Canadian communities with respect to freshwater on a scale from 0 to 100. The water availability component measures the renewable freshwater resources using the WSI thresholds as a benchmark: a score of 100 (highest) is assigned to any value over 1700 m^3^ capita^−1^ year^−1^ and 0 is assigned to any value below 500 m^3^ capita^−1^ year^−1^. The CWSI was developed by the Policy Research Institute (PRI) following the global application of the WPI in 2003 in which Canada ranked second out of 147 countries. The PRI maintained that Canada still had many challenges in water resources management including access to safe water among its rural indigenous communities, and considered an indicator analysis which better reflected these local challenges (PRI 2007).

Juwana et al. (2012) also noted that existing water sustainability indices (WPI, CWSI, Watershed Sustainability Index) had been developed in a context-specific manner to inform water resources sustainability and proposed a specialised West Java Water Sustainability Index (WJWSI) to address issues relevant to the sustainability of water resources in West Java, Indonesia. The WJWSI applies both the WSI and WTA as components within this multi-composite index. The WSI thresholds assess whether the availability of water in the study area is able to meet people’s absolute minimal water requirements, whereas the WTA ratios are adopted in the context of “water demand” to measure how much stress this demand puts on the water resources in the study area. The inclusion of WSI is specifically considered to be […] *extremely important for developing a water sustainability index* (Juwana et al. 2010, p. 1693).

Each of the Water Resources Sustainability indicators seeks to quantify characteristics of the human environment in order to measure water stress and scarcity. Similar to other holistic metrics, these approaches rely upon simplistic characterisations of human environments, and the weightings of components within each metric are subjective. Additionally, Water Resources Sustainability indicators can be based on highly localised community-level participatory approaches restricting their application at larger scales. Each also fails to move beyond MARR in defining physical freshwater availability.

#### Planetary boundaries

Recent discussion pertaining to the measurement of freshwater availability seeks to inform the planetary boundaries (PBs), proposed as the space within which humans can operate sustainably without threatening the resilience of the Earth system to persist in its Holocene-like state (Rockström et al. 2009; Steffen et al. 2015). Current debate (e.g. Gerten et al. 2015; Jaramillo and Destouni 2015b; Steffen et al. 2015) revolves around the uncertainty and robustness of assessments of consumptive freshwater use at the global scale and whether or not the proposed boundary of 4000 km^3^ year^−1^ has been reached. These deliberations represent a key departure from the scale of analyses of water scarcity reviewed above yet the PBs framework helpfully advances conceptual and computational estimation of the distribution of freshwater availability at smaller scales. First, the PBs framework explicitly recognises that freshwater resources and their use by humans at national or basins scales are inter-connected both in terms of their hydrological dynamics and their aggregated contributions to other Planetary Boundaries such as ‘Climate change’, ‘Biosphere integrity’ and ‘Land-system change’ (Steffen et al. 2015). Second, PBs research that focused on estimating consumptive freshwater use globally has served to advance the development of computational methods to estimate EWRs around the globe (e.g. Gerten et al. 2013). Third, PBs research has critically drawn attention to important feedbacks of human activity on consumptive freshwater use and downstream blue freshwater resources resulting from land-use change, irrigation and flow regulation (e.g. Destouni et al. 2013; Jaramillo and Destouni 2014, 2015a; Gerten et al. 2015). The influence of such local controls on consumptive freshwater use exposes, however, the limitations of the current PB debate that is focused on a global aggregate measure rather than the sustainability of local-scale freshwater withdrawals that comprise this global sum.

## Discussion

Metrics of water scarcity have evolved from simple thresholds of per capita freshwater availability based on MARR (e.g. WSI) to progressively more sophisticated metrics accounting for variability in demand (e.g. WTA), adaptive capacity (e.g. SWSI, Economic Water Scarcity), environmental water requirements (e.g. WSI_EWR_, Planetary Boundaries) and a range of social and environmental conditions (e.g. WPI, CWSI). The rationale for the WSI including its thresholds of water stress and scarcity was originally context-specific, based on the freshwater demand of an industrialised country in a semi-arid environment. Over the last three decades, however, the WSI and WTA have become globally applied standard metrics of water scarcity. Both rely upon assumptions that mask key factors affecting freshwater availability (e.g. inter- and intra-annual variations in river discharge) and are untested by evidence of whether computed water stress and scarcity are meaningful. We show additionally that characterisations of socio-economic dimensions of water scarcity embedded in more holistic metrics are subjective. Each of these key outcomes from our review is examined further below. We begin, however, by reviewing a common, fundamental misunderstanding between measured water scarcity and access to safe water that clearly separates SDG 6.4 from SDG 6.1: *By 2030, achieve universal and equitable access to safe and affordable drinking water for all*.

### Water scarcity is unrelated to access to safe water

**Fig. 3:**
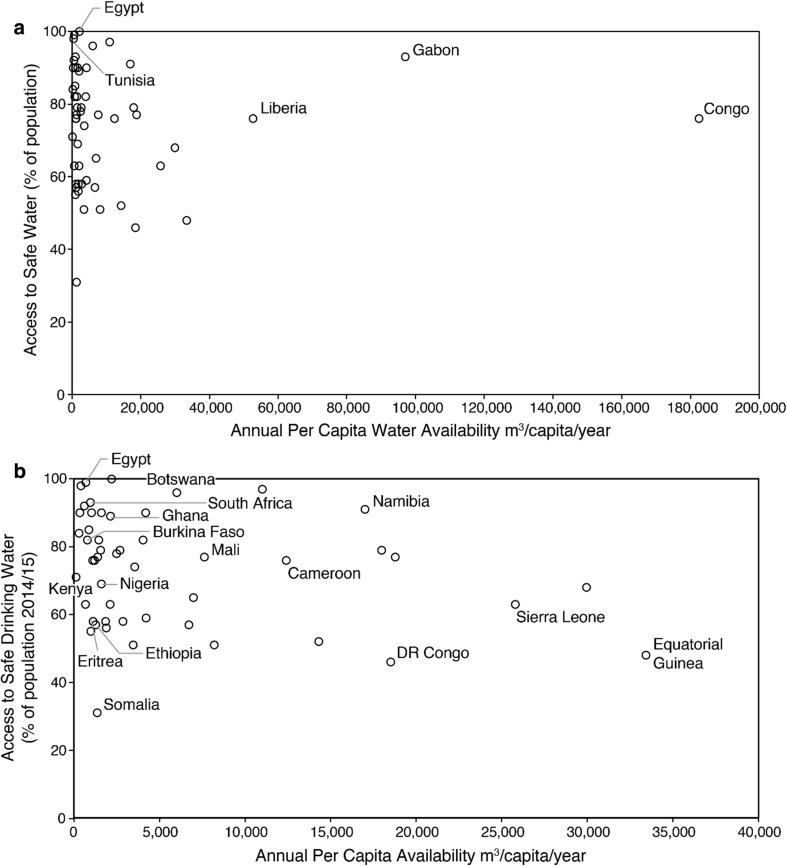
**a** Cross-plot relating national values of % access to safe water (World Health Organisation/Joint Monitoring Programme) to per capita freshwater availability across African 2014 (FAO AQUASTAT). **b** Cross-plot relating national values of % access to safe water (World Health Organisation/Joint Monitoring Programme) to per capita freshwater availability across Africa 2014 (FAO AQUASTAT), excluding extreme outliers in Fig. 3a

The World Water Assessment Programme (2003) report, “Water for People, Water for Life”, states *at present many developing countries have difficulties in supplying the minimum annual per capita water requirement of 1,700 cubic metres of drinking water necessary for active and healthy life for their people* (WWAP 2003, p. 10). This statement is problematic for two reasons. Firstly, it fails to recognise that the minimum annual per capita water requirement includes water used for industry and agriculture. Second, it represents a common misconception that access to safe drinking water depends upon freshwater availability, characterised by metrics of water scarcity. As shown in Fig. 3a, there is no statistically significant relationship (*r* = 0.03, *p* = 0.86) between access to safe water and per capita freshwater availability based on national-level statistics for African countries in 2014. Countries in North Africa such as Egypt and Morocco, which have low per capita freshwater availability and are defined by the WSI ‘water-scarce’ or ‘water-stressed’, report near-universal (>90%) access to safe drinking water. Excluding countries with a per capita freshwater availability exceeding 40 000 m^3^ year^−1^ (e.g. Congo, Gabon, Liberia), a weak *negative* association exists (*r* = −0.24, *p* = 0.09) between the proportion of the continent’s population that have access to safe water and annual amount of water availability per capita (Fig. 3b). As reported similarly by Chenoweth (2008), *[…] there is no evidence to support the statement of the World Water Assessment Programme* [above] *that countries require at least 1,700 cubic metres per capita to sustain a healthy and active life for their citizens*. Measured water scarcity is unrelated to measured coverage of access to safe water.

### Uncritical adoption of water scarcity metrics

The WSI was originally conceived in order to investigate the contribution of water scarcity to famines experienced in the Sudano-Sahel of Africa during the 1980s. Available data on freshwater resources at the time were sparse, and analyses employed L’vovich’s hydrogeological maps and limited observational records to make a preliminary assessment indication of where more detailed national studies should be conducted (Falkenmark 1989). The WSI was not specifically designed for continental and global-scale comparisons of water scarcity (Falkenmark 1989, p. 114). Indeed, the concept of a ‘water barrier’ (i.e. 2000 people/flow unit), derived from roundtable discussions in 1987, was contested from the outset (Falkenmark 1989) as engineers saw technology as a means of increasing supply whereas economists argued that demand for water can be controlled through pricing. Proposed thresholds of the water stress and water scarcity in the WSI (Table 1) recognised, however, limitations in both technology and pricing to influence freshwater supply and demand in Sudano-Sahelian Africa at the time (Falkenmark 1989). Gardner-Outlaw and Engelman (1997, p. 11), key proponents of the WSI, acknowledged that: *It would be, inappropriate, therefore, to propose any precise levels as absolute thresholds of water scarcity, or insist that they apply equally to all countries*. Consequently, the basis for the endorsement of water stress and scarcity thresholds in the WSI and WTA for continental-scale and global-scale applications (e.g. WWAP 2003, p. 10; Wada 2013; Schewe et al. 2013) remains unclear.

**Fig. 4:**
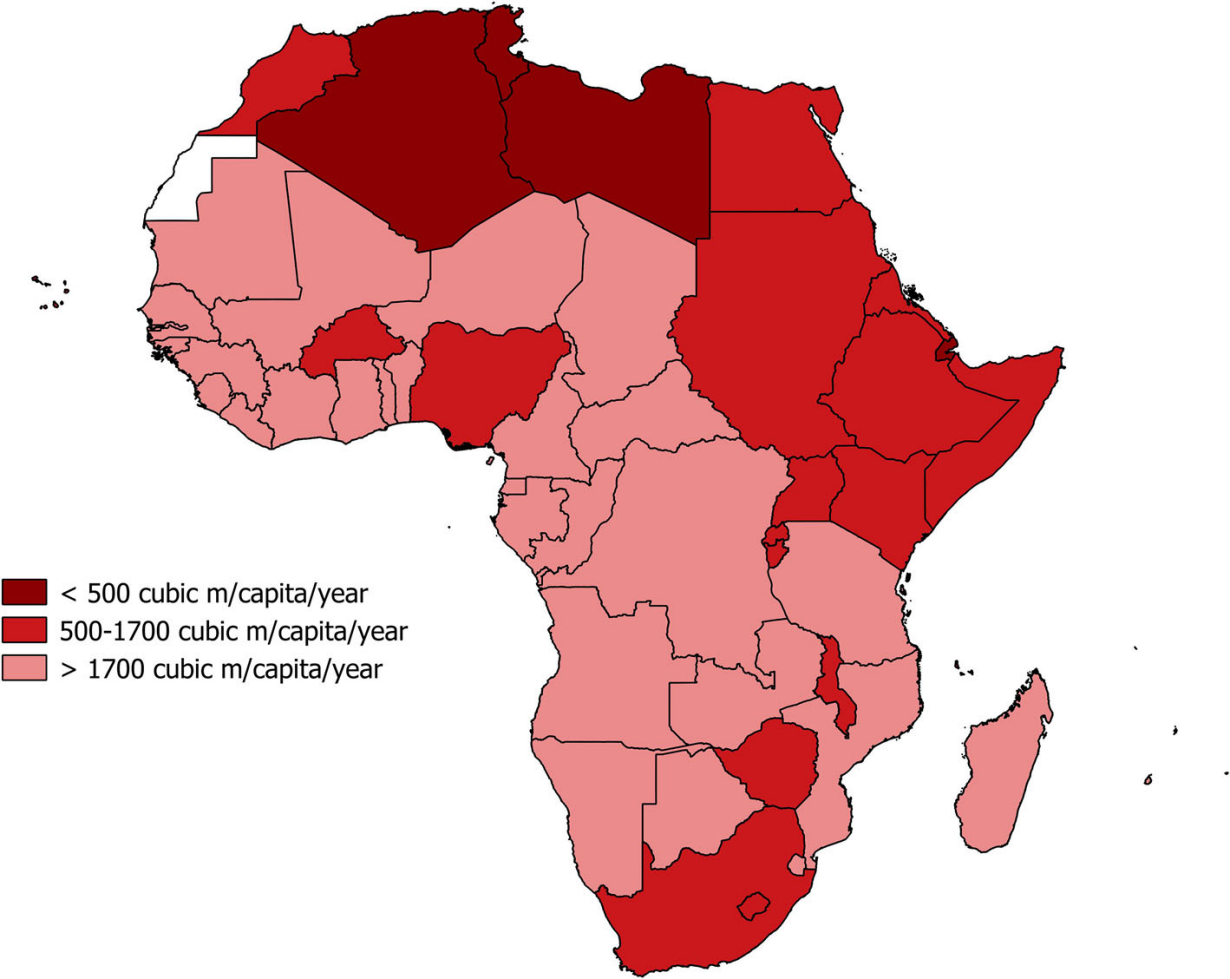
Map of national-scale water scarcity as defined by the water stress index (WSI) across Africa using data from the year 2014 (FAO AQUASTAT)

**Fig. 5:**
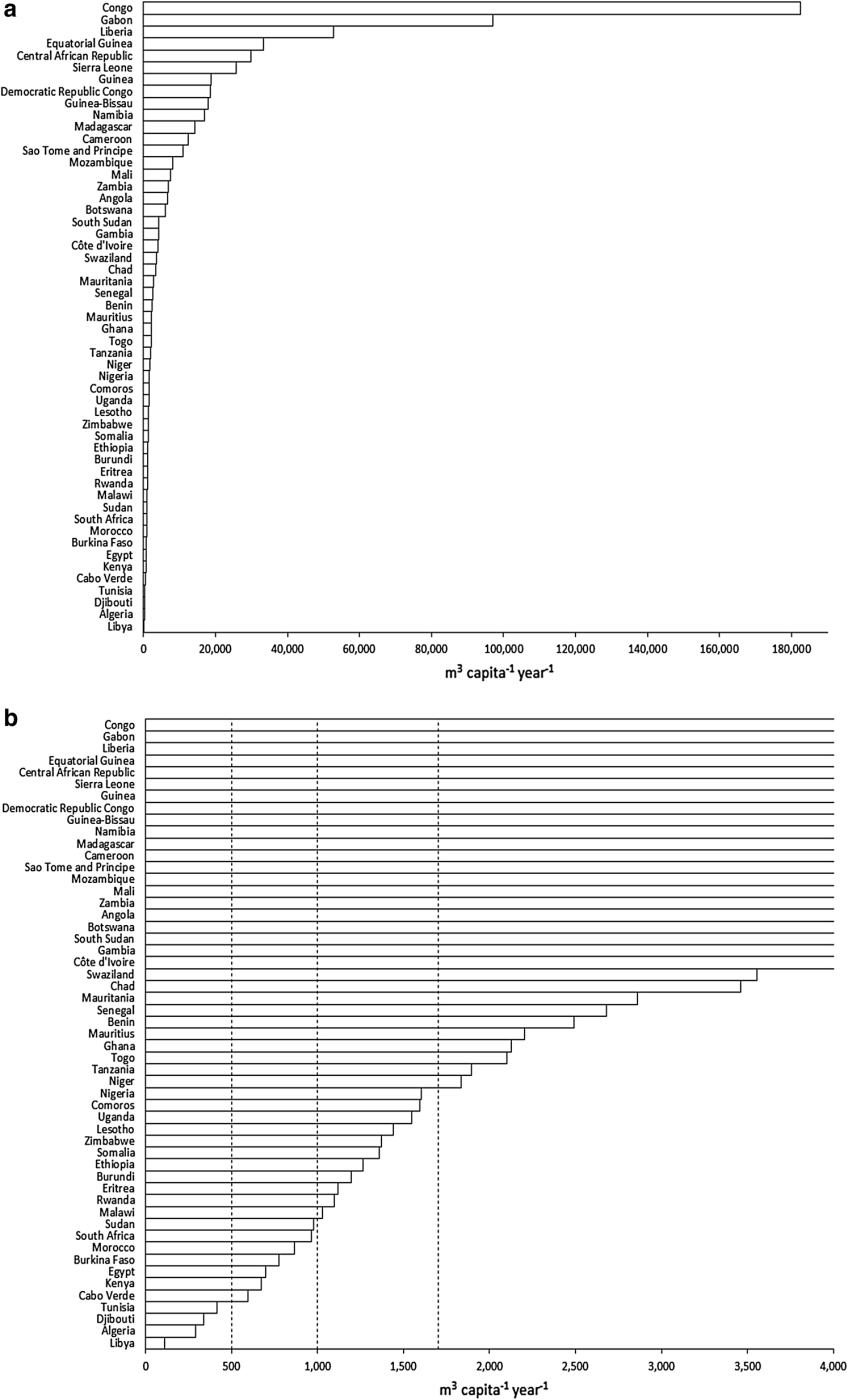
**a**, **b** National-scale per capita freshwater availability for African countries using data from the year 2014 (FAO AQUASTAT)

**Fig. 6:**
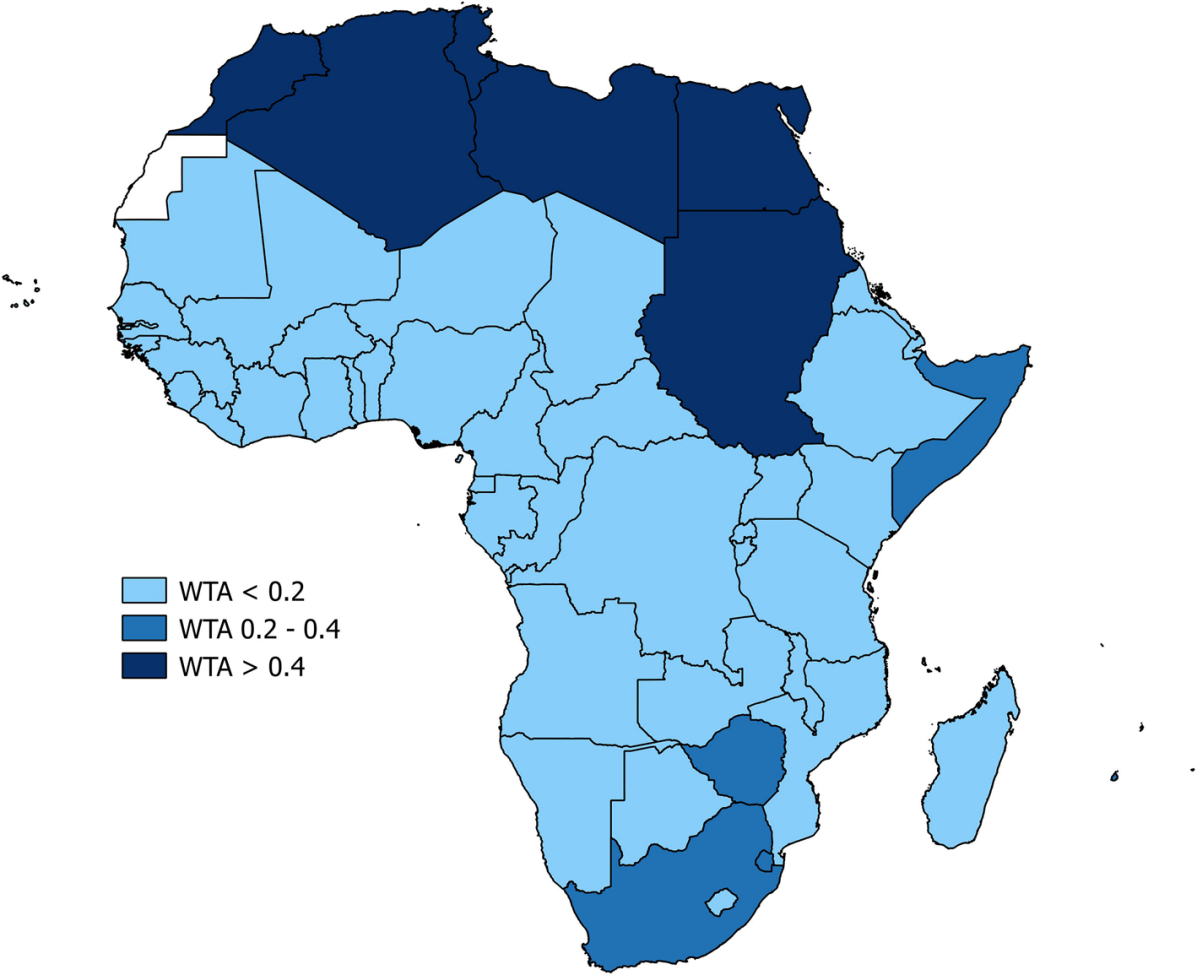
Map of national-scale water scarcity as defined by the withdrawal-to-availability (WTA) ratio across Africa using data from 2000 to 2002 (FAO AQUASTAT)

Application of the WSI and WTA to characterise water stress and water scarcity at national scales in Africa (Figs. 4, 5, 6; Table 2) produces differing outcomes. Most countries in Africa are characterised as water sufficient by both metrics yet twice as many countries are defined as “water scarce” or “water stressed” using the WSI than the WTA. 11 of 53 countries are defined as “water scarce” using the WSI (2014 data), whereas just 6 countries are characterised as “water scarce” by WTA. There are also some notable inconsistencies including Kenya, which is defined as “water scarce” according to the WSI (674 m^3^ capita^−1^ year^−1^) but deemed “water sufficient” using the WTA ratio (10%). Indeed, the uncritical adoption and application of the WSI and WTA to define freshwater availability in Africa are unreconciled to what is known of freshwater demand and supply; the latter is discussed in the next section "Misrepresentation of renewable freshwater resources" by MARR, whereas the former is considered here. First and foremost, the percentage of arable land that is irrigated in Africa remains low, <5% in Sub-Saharan Africa according to Giordano (2006) though this assessment may not account fully for small-scale irrigators across this region (Villholth et al. 2013). As rain-fed crop production dominates food production, the assumption embedded in the applied WSI (Table 1), that agricultural and industrial freshwater demand amounts to 20 times domestic demand, is indefensible. Further, the assumption that domestic demand is 100 LCPD is exaggerated. Although domestic consumption of this magnitude may very well be desirable, particularly for hygiene purposes (e.g. Cairncross 2003), a multi-site longitudinal analysis of domestic water use in East Africa (Thompson et al. 2001) indicates that per capita, domestic consumption is less than half the assumed volume and is declining rather than rising (Table 3).

**Table 2:** Differences in WSI and WTA of African countries (AQUASTAT)

Country	WSI (2014)	WSI (2002)	WTA (2002)
Algeria	Absolute Water Stress	Absolute water stress	Severely stressed
Angola	Sufficient	Sufficient	No stress
Benin	Sufficient	Sufficient	No stress
Botswana	Sufficient	Sufficient	No stress
Burkina Faso	Water Stress	Water scarcity	No stress
Burundi	Water Scarcity	Sufficient	No stress
Cabo Verde	Water Stress	Water stress	No stress
Cameroon	Sufficient	Sufficient	No stress
Central African Republic	Sufficient	Sufficient	No stress
Chad	Sufficient	Sufficient	No stress
Comoros	Water Scarcity	Sufficient	No stress
Congo	Sufficient	Sufficient	No stress
Côte d’Ivoire	Sufficient	Sufficient	No stress
Democratic Republic of the Congo	Sufficient	Sufficient	No stress
Djibouti	Absolute Water Stress	Absolute water stress	No stress
Egypt	Water Stress	Water stress	Severely stressed
Equatorial Guinea	Sufficient	Sufficient	No stress
Eritrea	Water Scarcity	Sufficient	No stress
Ethiopia	Water Scarcity	Sufficient	No stress
Gabon	Sufficient	Sufficient	No stress
Gambia	Sufficient	Sufficient	No stress
Ghana	Sufficient	Sufficient	No stress
Guinea	Sufficient	Sufficient	No stress
Guinea-Bissau	Sufficient	Sufficient	No stress
Kenya	Water Stress	Water stress	No stress
Lesotho	Water Scarcity	Water scarcity	No stress
Liberia	Sufficient	Sufficient	No stress
Libya	Absolute Water Stress	Absolute water stress	Severely stressed
Madagascar	Sufficient	Sufficient	No stress
Malawi	Water Scarcity	Water scarcity	No stress
Mali	Sufficient	Sufficient	No stress
Mauritania	Sufficient	Sufficient	No stress
Mauritius	Sufficient	Sufficient	Water stress
Morocco	Water Stress	water stress	Severely stressed
Mozambique	Sufficient	Sufficient	No stress
Namibia	Sufficient	Sufficient	No stress
Niger	Sufficient	Sufficient	No stress
Nigeria	Water Scarcity	Sufficient	No stress
Rwanda	Water Scarcity	Water scarcity	No stress
Sao Tome and Principe	Sufficient	Sufficient	N/A
Senegal	Sufficient	Sufficient	No stress
Sierra Leone	Sufficient	Sufficient	No stress
Somalia	Water Scarcity	Sufficient	Water stress
South Africa	water stress	water scarcity	Water stress
South Sudan	Sufficient	n/a	No stress (2011)
Sudan	Water Stress	n/a	Severely stressed (2011)
Swaziland	Sufficient	Sufficient	Water stress
Togo	Sufficient	Sufficient	No stress
Tunisia	Absolute Water Stress	Absolute water stress	Severely stressed
Uganda	Water Scarcity	Sufficient	No stress
United Republic of Tanzania	Sufficient	Sufficient	No stress
Zambia	Sufficient	Sufficient	No stress
Zimbabwe	Water Scarcity	Water scarcity	Water stress

**Table 3:** Per capita domestic water use in East Africa (Thompson et al. 2001)

Piped house holds			Unpiped households (urban)		Unpiped households (rural)	
Country	Litres/capita/day		Litres/capita/day		Litres/capita/day	
	1997	1966–1968	1997	1966–1968	1997	1966–1968
Kenya	47.4	121.6	22.9	11.3	22.3	8.2
Tanzania	80.2	141.8	25.1	17.8	16.0	10.1
Uganda	64.7	108.3	23.5	14.3	14.8	11.5

The continued, widespread application of WSI and WTA to measure water scarcity across Africa and beyond derives, in part or in whole, from their ease of application and comprehension (Rijsberman 2006). Little attention has been paid as to whether their application is meaningful. Savenije (1999) argues that *[*…*there] is definitely a need to develop water scarcity indicators that give a more reliable image of the water stress that is experienced in different parts of the world. A proper indicator should take into account all the renewable resources (including green water), should consider temporal and spatial variability and the influence of climate, should distinguish between primary and secondary needs and should use an objective key for the distribution for water resources among riparians*. At the 2014 World Water Week in Stockholm, Malin Falkenmark herself argued that the time is ripe for critically examining a move beyond the continued application of the WSI (Falkenmark, pers. comm.).

### Misrepresentation of renewable freshwater resources by MARR

The WSI, WTA and more holistic metrics compute renewable freshwater resources based on observations or simulations of MARR. As highlighted above in "Water stress index (WSI)" in section, MARR represents average ‘blue water’ resources that derive from the difference between mean precipitation and actual evapotranspiration assuming changes in freshwater storage are negligible (Eqs. 1, 2). The widespread continuous use of a singular value to characterise freshwater resources masks not only the temporal variability in freshwater resources but also the sources of this freshwater. Sub-Saharan Africa, for example, experiences substantial variations in both seasonal and inter-annual rainfall that produce the most variable river discharge in the world (McMahon et al. 2007). The fundamental characteristics of water resources in this region are typically defined by this variability, which is masked through the use of MARR. Further, groundwater resources which are not explicitly represented in MARR and considered only in so far as they contribute to river discharge, are estimated to amount to more than 100 times MARR in many countries in Africa (MacDonald et al. 2012). The distributed nature of groundwater in both sustaining river discharge during dry periods and enabling access to freshwater spatially to areas away from river channels is similarly obscured through the use of MARR. MARR further disregards ‘green water’ (i.e. soil water) which, as outlined above, sustains almost all food production in Sub-Saharan Africa. Consequently, water scarcity assessments employing MARR not only overestimate demand but also underestimate renewable freshwater resources (Taylor 2009). Indeed, the importance of explicitly considering the use of ‘green water’ in determining (consumptive) freshwater use of blue water resources is now well recognised in the Planetary Boundaries framework (e.g. Jaramillo and Destouni 2015b; Gerten et al. 2015).

Recent progress has been made in characterising intra-annual variability in freshwater resources by examining the relationship between freshwater availability and demand on a monthly time-step (Hanasaki et al. 2008b; Wada et al. 2011, 2014; de Graaf et al. 2014; Mekonnen and Hoekstra 2016); these analyses reveal previously undetected (masked) water-stressed areas. Alcamo et al. (2007) propose the consumption-to-Q_90_ ratio in which “consumption” is taken as the average monthly volume of water evaporated and “Q_90_” is a measure of the monthly discharge that occurs under dry conditions (i.e. when monthly discharge exceeds the Q_90_ value for 90% of the time). Q_90_ was subsequently applied by Wada and Bierkens (2014) in the Blue Water Sustainability Index, which also incorporates non-renewable groundwater use, to account for environmental streamflow. Brauman et al. (2016) recently developed the Water Depletion Indicator which measures the fraction of annual average renewable water (i.e. available surface and groundwater) which is consumptively used by human activities within a watershed, both annually, seasonally and in dry years. Critically, this study highlights the importance of seasonality, showing that watersheds that appear to be moderately depleted on an annual time-scale can be heavily depleted at seasonal time-scales or in dry years.

**Fig. 7:**
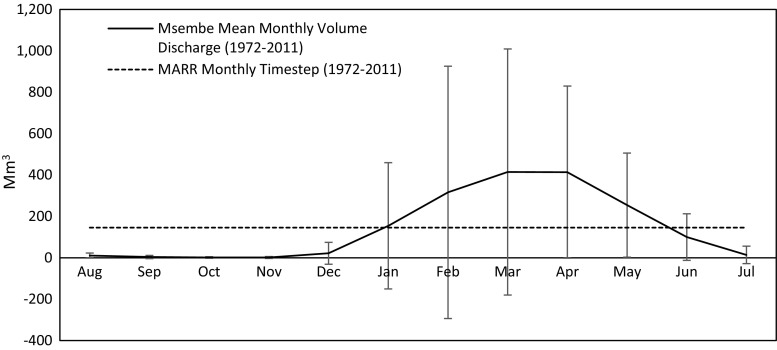
Mean monthly discharge (1972–2011) of the Great Ruaha River at Msembe, Tanzania (gauged area: 23 520 km^2^); *vertical bars* represent standard deviation in mean monthly river discharge; the *dashed line* indicates MARR expressed on a monthly time-step

Seasonal variability in river discharge is often substantial in semi-arid regions but masked through the estimation of renewable freshwater resources in terms of MARR. For example, in the Great Ruaha River Catchment of southwestern semi-arid Tanzania, rainfall and river discharge are seasonal, comprising a short but intense wet season (December–March) and long dry season. Mean monthly river discharge (Fig. 7) can as high as 414 Mm^3^ (equivalent to 160 m^3^ s^−1^) yet for five months of the year (July–November) mean monthly discharge is just 6 Mm^3^ (~4 m^3^ s^−1^). Indeed, recently, there have been several occasions when river discharge has ceased at the end of the dry season (Kashaigili 2008). The computed value of MARR, represented as a mean monthly value in Fig. 7 (146 Mm^3^ or 55 m^3^ s^−1^), obscures the fact that for nearly half of the year average renewable freshwater resources are one-tenth of this value. Consequently, all metrics of water scarcity that employ MARR to define renewable freshwater resources distort actual freshwater availability for much of the year in regions like the Great Ruaha Catchment.

### Subjective quantifications of socio-economic factors influencing water scarcity

The emergence of holistic metrics of water scarcity recognises that socio-economic, environmental and political factors can influence the occurrence of shortages in the availability of renewable freshwater relative to demand. We question, however, whether these factors can be meaningfully quantified. Scientific legitimacy is often sought through quantification. Although objectivity and neutrality may be implied through the impersonality of numbers, subjectivity is often embedded in the design of multi-component indicators including choices about which variables or parameters are included or excluded. Further, during the development of quantitative, multi-component metrics, procedures such as normalisation and weighting of variables employ subjective decisions (Freudenberg 2003; Nardo et al. 2005), for which there are rarely clear or formal declarations. The final step in multi-component metrics is aggregation, enabling direct comparisons of multiple variables transformed into a score-based outcome. A review of the normalisation, weighting and aggregation approaches taken in the formulation of the top 11 most globally applied Sustainable Development indices, revealed no consistent application of these principles yet all of these indices are generally accepted as being ‘scientifically robust’ (Böhringer and Jochem 2007).

Aside from the technical challenges of objectively normalising, weighting and aggregating a multi-component metric, quantification of the human environment in existing water scarcity metrics reduces contextual complexities to a narrow set of assumed determinants of water scarcity such as HDI and GDP (Gross Domestic Product). As argued by Zeitoun et al. (2016), such approaches ultimately underplay issues of equity and power. Indeed, ‘reductionist’ approaches oversimplify and thereby misrepresent determinants of water scarcity that could be better explored through a more integrative *Pathways Approach* (Leach et al. 2007), for example, which embraces diversity in society and the environment, and is able to consider freshwater resources beyond MARR. In this context, we argue that definitions of water scarcity might be best restricted to physical descriptions, which set a physical context within which a range of development pathways from the human environment (e.g. virtual water trade) can be considered to alleviate water scarcity (Hoekstra & Mekonnen 2012).

## Concluding discussion: Redefining water scarcity in terms of storage

Current assessments of water stress and scarcity commonly employ a metric, the WSI, conceived more than 30 years ago to explore potential linkages between freshwater availability and famines in the Sudano-Sahel of Africa. The simplicity of the WSI, which has contributed to its widespread adoption, fundamentally misrepresents both freshwater resources and demand in regions such as Sub-Saharan Africa (SSA). The WSI, WTA ratio and more holistic metrics reviewed here define renewable freshwater resources in terms of the singular measure of mean annual river runoff (MARR), which denies variability in freshwater resources and disregards both ‘green water’ (i.e. water embedded in plants and soil) and freshwater stored as groundwater or in lakes, dams and reservoirs. Indeed, the persistent focus on defining water scarcity strictly in terms of freshwater fluxes of supply and demand via metrics such as the WSI, WTA and their more recent manifestations is surprising since adaptive strategies to perennial or seasonal shortages in water supply commonly seek to utilise and amplify freshwater storage.

Freshwater storage derived from large-scale infrastructure, such as dams and reservoirs, has been considered explicitly in a few flux-based assessments of water scarcity. Vörösmarty et al. (1997) incorporated reservoir routing schemes into their global hydrological model and Hanasaki et al. (2008b) incorporated the 452 largest reservoirs in the world with a storage capacity of over 10^9^ m^3^, which account for over 60% of global reservoir storage capacity (Hanasaki et al. 2008a). Similarly, Wada et al. (2014) updated the reservoir release simulations of Hanasaki et al. (2006) and van Beek et al. (2011) to incorporate the extensive Global Reservoir and Dams dataset (GranD) (Lehner et al. 2011) containing 6862 reservoirs with a total storage capacity of 6197 km^3^. These assessments mark an important advance on most flux-based calculations of water scarcity, but their restricted characterisation of freshwater storage to large dams and reservoirs still ignores the vital contribution of distributed freshwater storage provided by wells, small-scale dams, and rainwater harvesting (Taylor 2009; Rockström and Falkenmark 2015). The exclusion of groundwater storage is particularly problematic since it is the world’s largest distributed store of freshwater and globally supplies ~40% of all water used to sustain irrigation and access to safe water (Jarvis 2013; Taylor et al. 2013). Döll et al. (2012) estimate that groundwater accounted for more than a third (35%) of the freshwater withdrawn globally over the period from 1998 to 2002.

We propose three key changes to the characterisation of water scarcity. First, redefine water scarcity in terms of the freshwater storage, both natural and constructed, that is required to address imbalances in the intra- and inter-annual fluxes of supply and demand. Second, restrict the quantification of water scarcity to verifiable physical parameters describing freshwater supply and demand. Third, use physical descriptions of water scarcity as a starting point for participatory decision-making processes by which communities, districts, basins and nations resolve how to address quantified storage requirements.

**Fig. 8:**
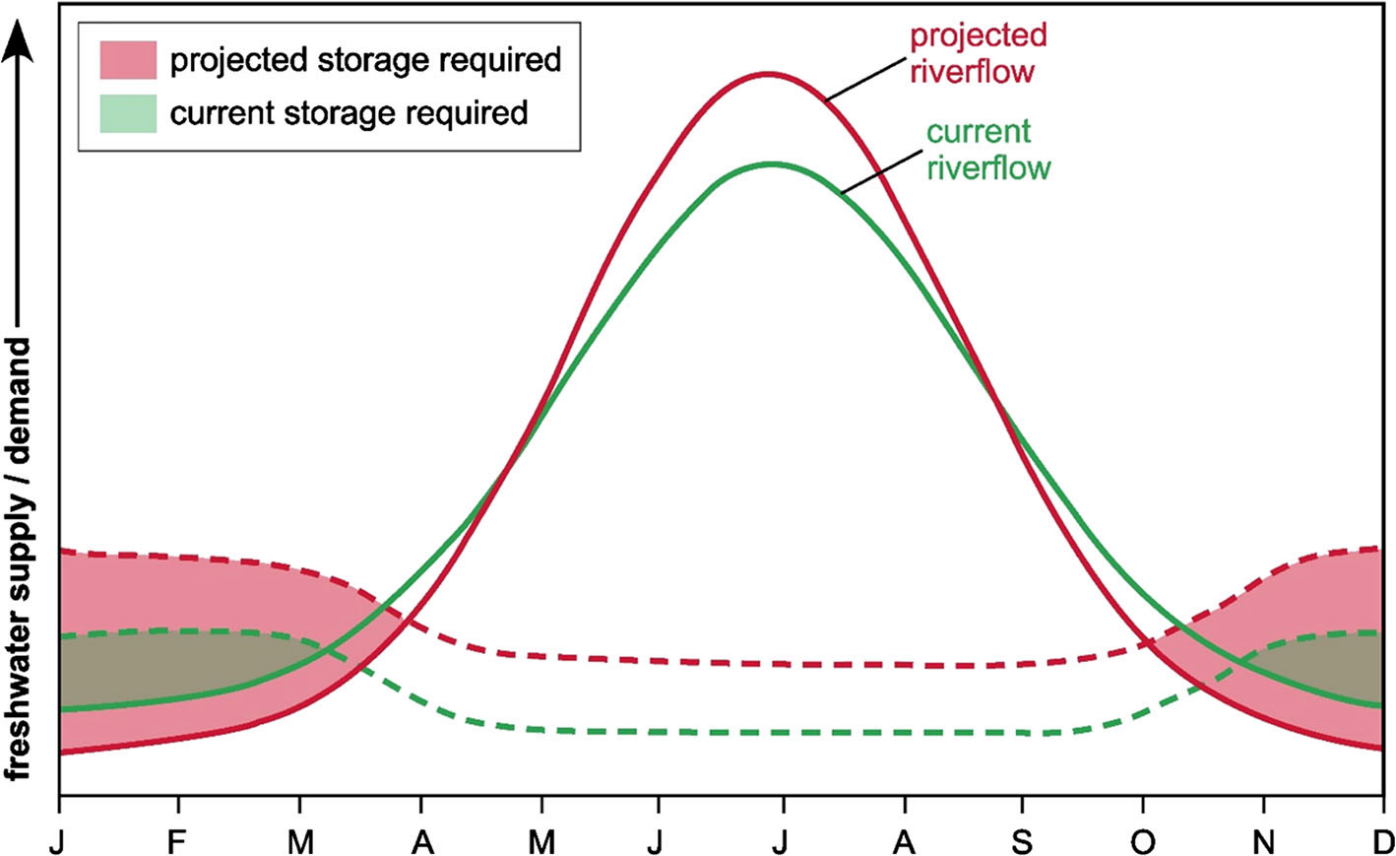
Conceptual representation of a river discharge regime under a monsoonal climate exhibiting a distinct (unimodal) intra-annual variability including the projected impact of the intensification of this river regime under climate change; and (2) intra-annual variability and change in freshwater demand (*dotted lines*) from all sectors including EWRs. *Shaded* areas mark periods when freshwater demand exceeds supply and quantify required access to freshwater storage. (*Reproduced with permission by Taylor*)

The first change explicitly considers intra- and inter-annual variability of freshwater supply and demand, which can control the magnitude and periodicity of water scarcity in a physical sense and translates this characterisation of water scarcity into an implementable, policy-relevant metric of storage requirements to be addressed. Figure 8 provides a conceptual representation^3^ of how estimated fluxes of freshwater supply and demand might be translated into a freshwater storage requirement. The figure depicts (1) a river discharge regime under a monsoonal climate exhibiting a distinct (unimodal) intra-annual variability including the projected impact of the intensification of this river regime under climate change; and (2) intra-annual variability and change in freshwater demand from all sectors including EWRs. Shaded areas in Fig. 8 mark periods when freshwater demand exceeds supply and quantify required access to freshwater storage. Water scarcity can then be defined physically as a measure of the extent to which required freshwater storage is available and used to inform adaptive responses reducing freshwater demand and/or increasing access to freshwater storage. Despite the availability of global databases for dams and reservoirs (Lehner et al. 2011), the process of quantifying available freshwater storage to include, among others, small-scale dams and renewable groundwater storage remains challenging. Substantial improvements in groundwater mapping have occurred (MacDonald et al. 2012) but robust estimates of groundwater recharge remain patchy and global-scale models of recharge remain largely uncalibrated and highly uncertain (Döll et al. 2016). It is also important to recognise that interventions reducing freshwater demand (e.g. increased use of ‘green water’) or increasing freshwater storage infrastructure (e.g. construction of dams or pumping wells) affect river discharge though the nature and magnitude of these effects can vary substantially. Destouni et al. (2013) estimate consumptive losses arising from dams and reservoirs globally to be 1257 km^3^ year^−1^. Although the use of distributed groundwater storage instead would theoretically reduce such losses, intensive groundwater abstraction has depleted available groundwater storage in some regions (Richey et al. 2015) while inducing greater recharge in others such as the Asian Mega-Deltas (e.g. Shamsudduha et al. 2011). Further, the conversion of native vegetation to crop cover has been observed to increase evapotranspirative losses in Sweden (Destouni et al. 2013) but to reduce these losses in the Sahel (Favreau et al. 2009). The second change we propose recognises the problematic quantification of human environments despite the fact that socio-economic and political factors play a dominant role in defining freshwater access (Zeitoun et al. 2016). This truism is well demonstrated here by the absence of a relationship between ‘water scarcity’ and ‘access to safe water’. The third change seeks to raise the utility of water scarcity determinations so that they inform a wide range of adaptive strategies, which are not restricted to large dams and reservoirs but include the use of renewable groundwater storage and rainwater harvesting as well as reducing freshwater storage requirements through the importation of food (i.e. virtual water trade) and increased water-use efficiencies.

## Electronic supplementary material

Below is the link to the electronic supplementary material.

Supplementary material 1 (PDF 138 kb)

## Footnotes

1 We define “freshwater demand” to mean water withdrawn to undertake human activities and that required to sustain Environmental Water Requirements. Freshwater demand is not to be confused with “consumption” or “consumptive uses of water”, which refer to a net water use wherein water is either not returned or its quality is altered to render it unusable.

2 Note that Ohlsson (2000) labels this as the *Hydrological Water Stress Index (HWSI);* it is equivalent to the Inverted Falkenmark WSI.

3 A superior but perhaps less intuitive version of this conceptual diagram based on a flow-duration curve considers inter-annual variability and is given in Fig. S1.
