# Supplementary material for: The measurement of water scarcity: Defining a meaningful indicator
Source: Ambio. 2017 Mar 15;46(5):513–31. doi: 10.1007/s13280-017-0912-z (PMC5547033; doi:10.1007/s13280-017-0912-z)
Supplement: Supplementary file 1 — Supplementary material 1 (PDF 138 kb) [file 13280_2017_912_MOESM1_ESM.pdf]

Ambio

Electronic Supplementary Material

This supplementary material has not been peer reviewed

Title: **The measurement of water scarcity: Defining a meaningful indicator**

Simon Damkjaer, Richard Taylor

Table S1. Overview of selected indicators as they relate to water scarcity and stress

Index	Reference	Scale	Description	Specific notes on water and scarcity components
**Water Requirements**				
Inverted Falkenmark	Falkenmark (1986)	National	Amount of people competing for 1,000,000 m^3 of water	Available freshwater resource is based on Mean annual river runoff
Traditional Falkenmark (Water Stress Index(WSI))	Falkenmark (1986, 1989)	National	Available freshwater resources per capita. See Table 1 for more details.	Available freshwater resource is based on Mean annual river runoff
Gleick	Gleick (1996)	National	Drinking = 5 Litres/person/day (l/p/d); sanitation = 20 l/p/d, bathing = 15 l/p/d, food preparation = 10 l/p/d; Total = 50 l/p/d. However, a global complete range is estimated to be 27 – 200 l/p/d and the paper finds "Falkenmark's 100 l/p/d falls well within the middle of this bracket".	Acknowledges that available sources of water differs across the world and can be culturally and societally determined.
Domestic Water Scarcity Index	Weligamage (1998)	Community	-	-
Social Water Stress Index	Ohlsson (2000)	National	Incorporates society's adaptive capacities to water scarcity	HDI-weighted measure of WSI (0-20 point scale) (See Table S2 for more information)
Cereal Input Index	Yang *et al*. (2003)	National (Africa and Asia only)	Stipulates that the correlation between volume of available freshwater and quantity of imported food can serve as a basis for a model which investigates net cereal import as a function of renewable water resources to serve as a water deficit indicator	Available freshwater resource is based on Mean annual river runoff
Water withdrawals to availability ratio (WTA ratio)	Raskin *et al*., (1997); Alcamo *et al*., (2003); Vorosmarty *et al*. (2005); Rijsberman (2006)	Local/National	Water use is defined as the sum of water withdrawals for domestic (D); industrial (I) and agricultural sectors (A) divided by total freshwater availability. If DIA withdrawals to availability is higher than 40%/0.4 there is water stress.	Available freshwater resource is based on Mean annual river runoff
Water Exploitation Index	Marcuell & Lallana (2003)	National	If mean total annual water abstractions (DIA) to total freshwater availability is over 40% there is severe water stress.	Available freshwater resource is based on Mean annual river runoff

Water Supply Stress Index	McNulty *et al*. (2010)	Local (Watershed) USGS Hydrological Unit datasets	Compares water demand to water supply (i.e. WTA ratio)	Available freshwater resource is based on Mean annual river runoff
**The Emergence of Holistic and Integrated metrics**				
Physical and Economical Water Index	Seckler *et al.*, (1998); IWMI (2008)	National	Physical water scarcity if >75% of river flows are withdrawn for DIA.<br><br>Economic scarcity if less than 25% of river flows are withdrawn for DIA but infrastructural development lacks investment.	Available freshwater resource is based on Mean annual river runoff
Water Poverty Index	Sullivan (2002); Lawrence *et al.*, *(2002); Sullivan* et al., *(2003); Lawrence* et al., *(2003); Fenwick (2010)*	National; later local/community/household.	The index clusters its components in five dimensions: 1) access to water; 2) water quantity; water quality and variability; 3) water uses for domestic, food and production purposes; 4) capacity for water management and; 5) environmental aspects.	WTA ratio component applied. WSI component is on a log-scale. For both WTA and WSI available freshwater resource is based on mean annual river runoff. Water for domestic purposes is set at 50 l/p/d.
Water Scarcity Index (WSCi): population growth impact on water resources availability	Asheesh *et al.*, (2007)	National	Measures magnitude of water deficit necessary to be returned into the natural system in order to sustain a balance between available water and water demand. Incorporates population growth rate, water availability, and domestic, industrial and ecological water usage	Available freshwater resource is based on Mean annual river runoff<br>Available Freshwater resources availability based on mean annual river runoff on a log-scale.
Water Stress Index (incorporating Environmental Water Requirements (WSI_{EWR})	Smakhtin *et al.*, (2004)	National	WTA ratio accounts for Environmental Water Requirements	

**Water Accounting Frameworks**				
Water Footprint	Hoekstra and Hung (2005)	National, river basin, local	The water footprint (WfP) is the virtual water (embedded water) in production of a good. A global trade can be visualised as an adaptive capacity	Originally available freshwater resources is based on mean annual river runoff
Life Cycle Assessment (LCA)	Pfister *et al.*, (2009)	Local and watershed	This assessment indicator uses the WSI_{EWR} combined with traditional Life Cycle Assessment approaches to measure environmental stresses.	The WTA ratio is applied as the hydrological component. A later study, using the same methods (Pfister and Bayer, 2014) recognises the importance of considering the temporal variability of freshwater availability. Available freshwater resource is based on mean annual river runoff
Water Impact Index (WII)	Bayart *et al.*, (2014)	Local	Adopts the LCA and WfP approaches with the aim to integrate issues that relate to water scarcity and quality in a single indicator in order to assess the water footprint of human uses of freshwater on the environment.	Water scarcity component of WII applies WSI_{EWR} methodology. Available freshwater resource is based on mean annual river runoff
**Water Sustainability Metrics**				
Watershed Sustainability Index	Chaves & Alipaz (2007)	Watersheds below 2,500 km^2	WSIndex incorporates hydrology (H), environment (E), life (L) and policy (P), each with the parameters "pressure, state and response".<br>The WSIndex value (ranged 0-1) is calculated as the average of HELP, all of which are also scored on a scale from 0-1. (See *Table S3* below for more information)	Water quantity parameter applies Falkenmark threshold of 1,700 m^3/capita/year and state water stress occurs under this level and applies five levels of per capita water availability in relation to multiples of this minimum standards. Available freshwater resource is based on Mean annual river runoff
Canadian Water Sustainability Index	PRI (2007)	Canadian Community Scale	Fifteen indicators are holistically integrated into the components of: Freshwater Resources; Ecosystem Health; Water Infrastructure; Human Health and Well-being; and Community Capacity	Available freshwater resources is based on Mean annual river runoff: Applies Falkenmark thresholds where a score is assigned of 100 is assigned to any value over 1,700 m^3/capita/year and 0 of 500 m^3/capita/year; indicator for supply serves as a proxy for the vulnerability of the community's freshwater supply by addressing the variability of surface water flows and/or trends in ground water reserves.<br><br>Demand indicator: demand on the resource is the amount of water annually allocated relative to the total amount of renewable fresh water.
Arab Water	Ali *et al.* (2008)	National	Four theme-based components were proposed to	Available freshwater resource is based on Mean annual river

Sustainability Index		(Arab region)	reflect a meaningful representation of the situation in the region: water crowding, dependency, scarcity and environmental sustainability.	runoff. The WSI is adopted to portray "water crowding" and the WTA ratio to measure water scarcity. This is done in the context of agricultural impact on water resources availability.
West Java Sustainability Index	Juwana (2012)	West Java, Indonesia	Composite indicator measuring components of Conservation; Water Use and Policy & Governance, incorporating water availability, demand and quality.	Available freshwater resources is based on Mean annual river runoff. The WSI is applied to portray water availability and the WTA to reflect water demand.
Water Resources Sustainability Evaluation Model	Kang & Lee (2011)		-	-
Aqueduct Water Risk Tool	Reigh *et al*. (2013); Gassert *et al*. (20130	National, Global	Publicly available global database that provides information on water-related risks worldwide for businesses, using three categories of indicators: Physical Risks: Quantity; Physical Risks: Quality; Reputational and Regulatory Risks	Available freshwater resource is based on mean annual river runoff WTA ratio approach to identify areas of water stress. The issue of seasonality in water supply between months is acknowledged as being a challenge.
Sustainability Gap Framework *In progress*	Ekins & Simon (2001).	National	Years to Sustainability is the time it will take to reach predefined sustainability goals. Years to Sustainability is the time it will take to reach a sustainability goal, which is calculated as the difference between a predefined sustainable level of impacts and the current level of environmental impacts from a specific pressure. .	*In progress*. Available freshwater resource is based on mean annual river runoff Severe stress occurs when WTA >40%.
**Water Security**				
Water Security Status Indicator approach	Norman *et al*. (2013)	Community	It's method rather than an indicator; integrates variables pertaining to water quantity and quality as they relate to aquatic ecosystems and human health.	Available freshwater resource is based on mean annual river runoff.
Climate vulnerability index	Sullivan & Meigh (2005)		Resource (R), Access (A), Capacity (C), Use (U), Environment (E) and; Geospatial (G) divided by eight risk factors	Available freshwater resource is based on mean annual river runoff
Governance and Climate Vulnerability index	Jubeh & Meigh (2012)		Combined Climate Vulnerability and Governance Index	Available freshwater resource is based on Mean annual river runoff . Applies the WSI.
Water Vulnerability Index	Sullivan (2011)	Municipal scale	supply-driven vulnerability (from water systems) (SDWV) and demand-driven vulnerability (from water users) (DDWV) dimensions are combined	Available freshwater resource is based on mean annual river runoff .

Bagmati River Basin Vulnerability Assessment	Babel *et al*. (2011)		Combination of water stress sub-index and adaptive capacity sub-index	Available freshwater resource is based on Mean annual river runoff. Applies the Falkenmark and WTA thresholds.
Arctic Water Resources Vulnerability index	Alessa *et al*. (2008)	Communities in circumpolar Arctic	An index to assess resilience toward changes in freshwater resources: 2 sub-indices: physical (quality and quantity) and social.	Physical water supply: measured via precipitation as average annual rainfall over 30 years. For the river flow indicator, the average annual runoff in the watershed and the Coefficient of Variance for that run-off over a 30-year time series are measured; seasonal variation in water supply, the difference in monthly maximum and minimum river discharge, normalised by the monthly mean river discharge is calculated in order to determine a measure for the intra-annual water supply variation.<br><br>Physical Water Supply: the ability to use infrastructure to continuously ensure that there is 20-100 l/capita/day available of water. AWRVI recognises the importance of the ability to store water to ensure resilience against times where natural supply may not be adequate to meet demands.
**Groundwater Sustainability Metrics**				
Groundwater Sustainability Infrastructure Index	Pandey *et al*. (2011)	National	existing knowledge, practices and institutions whose adequate strengthening helps to achieve groundwater sustainability is necessary infrastructure in evaluating progress in achieving groundwater sustainability	-
The International Hydrological Programme (IHP) Working Group on Groudwater Indicators	Lavapuro *et al*. (2008); Lamban *et al. (2011)*	National	measurable and observable data and information on groundwater quantity and quality and information on socio-economic and environmental matters	-
Social Sustainable Aquifer Yield	Molina *et al*., (2012)	Local	Introduced variable termed: Aquifer Social Yield (ASY); ASY is the social perception of the maximum acceptable aquifer exploitation, as derived at through stakeholder engagement at the local level	-

Table S2. The SWSI Ranking system (Ohlsson, 2000)

Index Ranking Intervals	HWSI/SWSI Score	Degree of Stress
0-5	> 1,700	Relative sufficient
6-10	< 1,700 – 1,000	Water Stress
11-20	< 1,000	Water Scarcity
20+	<500	Absolute Water Scarcity

Table S3. Watershed Sustainability Index parameters (Chaves and Alipaz, 2007)

**Indicator**	**State**	**Pressure**	**Response**
Hydrology	WSI variation<br>Variation in BOD5	Long term WSI<br>Long term BOD5	Water use/sewage efficiency
Environment	Environment Pressure Index	% basin with natural vegetation	Basin conservation
Life	Variation in per capita income	Basin HDI	Basin HDI Evolution
Policy	Variation in HDI Education parameter	Basin IWRM institutional capacity	Evolution of basin IWRM

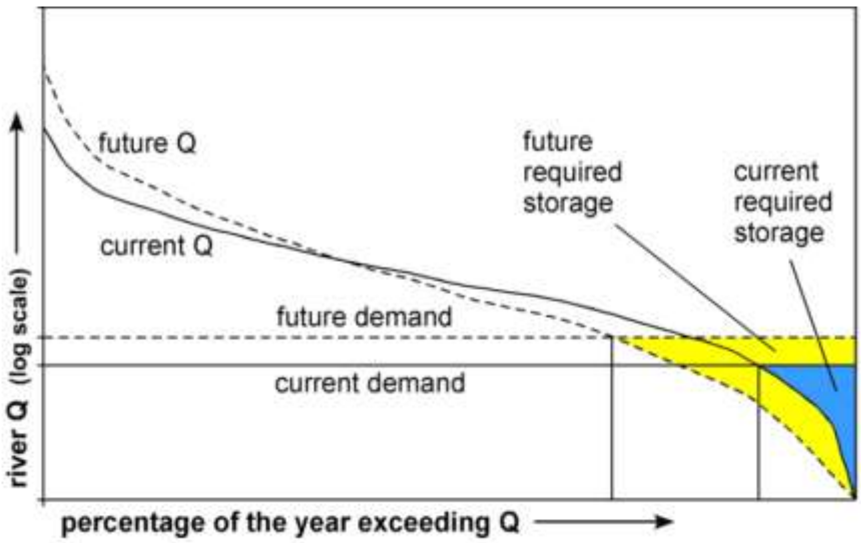

Figure S1. (1) Conceptual representation of a flow duration curve under a monsoonal climate exhibiting a distinct (unimodal) intra-annual variability including the projected impact of the intensification of this river regime under climate change; and (2) intra-annual variability and change in freshwater demand from all sectors including EWRs. Shaded areas in mark periods when freshwater demand exceeds supply and quantify required access to freshwater storage. (*Reproduced with permission by Taylor*).

References

Alessa, L., Kliskey, A., Lammers, R., Arp, C., White, D., Hinzman, L., Busey, R., (2008) "The Arctic Water Resource Vulnerability Index: An Integrated Assessment Tool for Community Resilience and Vulnerability with Respect to Freshwater" *Environmental* Management 42, 523–541

Ali, F., (2008) "Development of water stress index as a tool for the assessment of water stress areas in the metropolitan Jakarta" in: 16th Annual International Sustainable Development Research Conference, Hong Kong.

Allan, J.A., (1997) "Virtual water: a long term solution for water short Middle Eastern economies? *Occasional Paper 3*, School of Oriental and African Studies (SOAS), University of London.

Asheesh, M., (2003) "Allocating Gaps of Shared Water Resources (Scarcity Index): Case Study on Palestine-Israel", in: Shuval, H.H., Dweik (Eds.), *Water Resources in the Middle East: Israel-Palestine Water Issues - From Conflict to Cooperation*, Springer-Verlag, Berlin, 241–249.

Babel, M.S., Pandey, V.P., Rivas, A.A., Wahid, S.M., (2011) "Indicator-Based Approach for Assessing the Vulnerability of Freshwater Resources in the Bagmati River Basin, Nepal", *Environmental Management*, 48, 1044–1059

Bayart, J.B., Worbe, S., Grimaud, J. & Aoustin, E. (2014) The Water Impact Index: a simplified single-indicator approach for water footprinting, *International Journal of Lifecycle Assessment*, 19:1336-1344

Chaves, H.M.L. and Alipaz, S. (2007) "An Integrated Indicator Based on Basin Hydrology, Environment, Life and Policy: The Watershed Sustainability Index", *Water Resources Management*, 21:883-895.

Ekins, P. & Simon S. (2001)"Estimating Sustainability Gaps: Methods and Preliminary Applications for the UK and the Netherlands", *Ecological Economics*, 37, 1, 5-22.

Gassert, F, Landis, M., Luck, M., Reig, P., & Shiao, T. (2013) Aqueduct Global Maps 2.0, Working Paper, Washington D.C.: World Resources Institute.

Gleick, P.H., (1996) "Basic Water Requirements for Human Activities: Meeting Basic Needs", *Water International* 21, 83–92.

Hoekstra AY, Hung PQ (2005) Globalisation of water resources: International virtual water flows in relation to crop trade. Global Environmental Change 15(1):45–56

Jubeh, G. & Mimi, Z. (2012) "Governance and Climate Vulnerability Index", *Water Resources Management*, 26, 4147–4162.

Kang, M.-G. & Lee, G.-M. (2011) "Multicriteria Evaluation of Water Resources Sustainability in the Context of Watershed Management", *JAWRA Journal of the American Water Resources Association*, 47, 813–827.

Lambán, L.J., Martos, S., Rodríguez-Rodríguez, M. & Rubio, J.C. (2011) "Application of groundwater sustainability indicators to the carbonate aquifer of the Sierra de Becerrero (Southern Spain)", *Environmental Earth Sciences*, 64, 1835–1848.

Lavapuro, M., Lipponnen, A., Artimo, A. & Katko, T.S. (2008) "Groundwater sustainability indicators: testing with Finnish data", *Boreal Environment Research*, 13, 381-402.

Marcuello, C., Lallana, C., (2003) "Water Exploitation Index explained", *European Environmental Agency*.

McNulty, S.G., Sun, G., Myers, J.M., Cohen, E. & Caldwell, P. (2010) "Robbing Peter to Pay Paul: tradeoffs between ecosystem carbon sequestration and water yield", *Watershed Management*, 23–27.

Molina, J.-L., Martos-Rosillo, S., Martín-Montañés, C. & Pierce, S. (2012), "The Social Sustainable Aquifer Yield: An Indicator for the Analysis and Assessment of the Integrated Aquifers Management", *Water Resources Management*, 26, 2951–2971.

Norman, E.S., Dunn, G., Bakker, K., Allen, D.M.. & Cavalcanti de Albuquerque, R. (2013) "Water Security Assessment: Integrating Governance and Freshwater Indicators", *Water Resources Management*, 27, 535–551.

Ohlsson, L. (2000) "Water conflicts and social resource scarcity", Physics and Chemistry of the Earth, Part B: 791 Hydrology, Oceans and Atmosphere, 25, 213–220.

Pandey, V.P., Shrestha, S., Chapagain, S.K. & Kazama, F. (2011) "A framework for measuring groundwater sustainability", *Environmental Science & Policy*, 14, 396–407.

Pfister, S. & Bayer, P. (2014) "Monthly Water Stress: Spatially and temporally explicit consumptive water footprint of global production, *Journal of Cleaner Production*, 73:52-62.

Pfister, S., Koehler, A. & Hellweg, S. (2009) "Assessing the Environmental Impacts of Freshwater Consumption in LCA", *Environmental Science & Technology*, 43, 4098–4104.

Raskin, P. (1997) "Water Futures: Assessment of long-range patterns and problems. Comprehensive Assessment of the Freshwater Resources of the World", Stockholm Environment Institute.

Reigh, P., Shiao, T. & Gassert, F. (2013) "Aqueduct Water Risk Framework", Washington, DC: World Resources Institute, Working Paper.

Sullivan, C. (2011) “Quantifying water vulnerability: a multi-dimensional approach”, *Stochastic Environmental Research and Risk Assessment*, 25, 627–640.

Sullivan, C. & Meigh, J. (2005) “Targeting attention on local vulnerabilities using an integrated index approach: the example of the climate vulnerability index”, *Water science & technology*, 51, 69–78.

Sullivan, C., Meigh, J. & Giacomello, A.M. (2003) “The water poverty index: development and application at the community scale”, *Natural Resources Forum*, 189–199.

Weligimage, P. (1998) “Developing a domestic water scarcity index in a multiple water source situation”, Paper prepared for the national conference on Status and Future Direction of Water Research, Sri Lanka, Colombo, IWMI.

Yang, H., Reichert, P., Abbaspour, K.C. & Zehnder, A.J., (2003) “A water resources threshold and its implications for food security”, *Environmental science & technology*, 37, 3048–3054.
